# The Emerging Key Role of the mGluR1-PKCγ Signaling Pathway in the Pathogenesis of Spinocerebellar Ataxias: A Neurodevelopmental Viewpoint

**DOI:** 10.3390/ijms23169169

**Published:** 2022-08-15

**Authors:** Qin-Wei Wu, Josef P. Kapfhammer

**Affiliations:** Department of Biomedicine, Institute of Anatomy, University of Basel, 4056 Basel, Switzerland

**Keywords:** spinocerebellar ataxias, Purkinje cell dendritic development, PKCγ, RGS8, STK17B, mGluR1, INPP5A

## Abstract

Spinocerebellar ataxias (SCAs) are a heterogeneous group of autosomal dominantly inherited progressive disorders with degeneration and dysfunction of the cerebellum. Although different subtypes of SCAs are classified according to the disease-associated causative genes, the clinical syndrome of the ataxia is shared, pointing towards a possible convergent pathogenic pathway among SCAs. In this review, we summarize the role of SCA-associated gene function during cerebellar Purkinje cell development and discuss the relationship between SCA pathogenesis and neurodevelopment. We will summarize recent studies on molecules involved in SCA pathogenesis and will focus on the mGluR1-PKCγ signaling pathway evaluating the possibility that this might be a common pathway which contributes to these diseases.

## 1. Introduction

Spinocerebellar ataxias (SCAs) are a heterogeneous group of autosomal dominantly inherited progressive disorders with degeneration and dysfunction of the cerebellum [1,2,3]. By now, more than 40 genetically distinct subtypes of SCA have been identified. The genetic background of SCAs can be classified into two groups: Group I representing repeat expansion SCAs, such as SCA1 and SCA2 which are caused by dynamic repeat expansion mutations, typically polyglutamine repeat expansions, and Group II representing conventional mutation SCAs (non-repeat expansion SCAs), which are caused by nonsense mutations, missense mutations, deletions or insertions, such as SCA5 or SCA14. In general, signs and symptoms can develop in patients with SCA at any time from childhood to late adulthood, but adult-onset is the most common [1,2,3]. Previous studies have shown that in Group II conventional mutation SCAs the age of onset is earlier than in SCAs due to polyglutamine repeat expansions [1,2]. The clinical features of all SCAs include progressive loss of balance and coordination, accompanied by slurred speech. The mobility and communicative skills of individuals with SCA are reduced and many SCAs lead to premature death. As the name suggests, pathological changes of SCAs occur primarily in the nervous system and the cerebellum is the principal target. Purkinje cell degeneration, which leads to cerebellar atrophy, occurs in most SCAs [1,2,3]. The pathways causing degeneration or loss of Purkinje cells are complex. Dysregulation of gene expression is an acknowledged characteristic of several SCAs and has been proposed to trigger the pathogenesis of SCAs [4,5]. Several proteins which can cause SCAs when they contain mutations are recognized as key factors associated with the regulation of gene expression and contribute to gene regulation on the transcriptional level. In some representative types of SCAs caused by polyglutamines, such as SCA1 and SCA7, the causative proteins are ataxins which interact with transcriptional regulators and indirectly affect gene expression especially for developmental processes [6,7,8,9]. Some SCA causing genes are directly responsible for the regulation of gene expression, e.g., TBP in the case of SCA17. The TATA-Box binding protein (TBP) is a general transcription factor and mutants of TBP with CAG repeat expansion result in SCA17 [10,11]. Hence, these studies suggest that mutated proteins of SCAs can affect gene expression directly or indirectly by changing the activity of signaling proteins resulting in a dysregulation of transcription. 

Some studies have focused on the analysis of the gene expression on the global transcriptional level by the use of animal or cellular models and aimed to identify genes with an altered expression in SCAs [12,13,14]. In this review article, we will specifically focus on the molecules linking Purkinje cell development to SCAs and elaborate the function of these molecules that are causing SCA and at the same time play a role in the development of Purkinje cells. Furthermore, we will review recently identified key molecules dysregulated in different SCAs and discuss how they participate in the emerging shared pathogenic pathways in the SCAs, in particular the mGluR1-PKCγ signaling pathway.

## 2. Increasing Evidence Linking Purkinje Cell Dendritic Development to SCAs

Purkinje cells are cells which have a large and highly branched dendritic tree. Many molecules participating in the regulation of different stages of Purkinje cell development have been identified, including dendritic growth, differentiation, and maintenance. Examples for these molecules are Beta-III spectrin, PKCγ, TRPC3 and mGluR1. The genes (*SPTBN2*, *PRKCG*, *TRPC3* and *GRM1*) encoding these molecules have also been identified as causative genes of SCAs and are probably associated with pathogenesis of SCAs. Here we will briefly review the current information about these molecules with respect to Purkinje cell dendritic development and their involvement in SCA pathology.

### 2.1. SCA5

SCA5 is caused by gene mutations of *SPTBN2* encoding Beta-III spectrin protein [15,16,17]. The mutant of Beta-III spectrin protein was strongly expressed in Purkinje cells by immunofluorescence staining in a SCA5 mouse model with a phenotype of progressive cerebellar degeneration [18]. Beta-III spectrin has been identified to play a critical role in the organization of the dendritic tree and the development of dendritic spines of Purkinje cells. Beta-III spectrin knockout mice have defects of the ordered dendritic arborization, particularly a loss of monoplanar organization, a decreased dendritic diameter, a reduction of the density of dendritic spines and a reduced number of synapses in Purkinje cells. Purkinje cells from Beta-III spectrin knockout mice also show strongly reduced dendritic areas compared to wildtype cells in dissociated cultures [17]. 

### 2.2. SCA6

The alteration of calcium channel conductance is believed to be an important factor in the pathology of SCAs. SCA6, which is caused by a CAG repeat expansion in the *CACNA1A* gene, encoding the voltage-gated calcium channel subunit alpha 1A, belongs to the Group I type of SCA [19], but there are also a number of SCA6 patients with point mutations of this gene [20,21]. Overlapping phenotypes have been reported for SCA6 caused by missense mutations, episodic ataxia type 2 (EA2) and familial hemiplegic migraine (FHM). The causative gene of EA2 and FHM is also *CACNA1A*, previously called *CACNL1A4* [22]. A mutation of the *CACNA1*A gene, the causative gene of SCA6, was also reported in the tottering mouse. In this mouse model, a reduction of dendritic development was observed [23]. The *CACNA1A* gene can functionally encode two proteins: the α1A subunit of P/Q-type voltage gated calcium channel and α1ACT, a transcription factor, encoded by a second cistron in the *CACNA1A* gene. Recent studies have found that α1ACT is involved in the regulation of genes associated with the function of Purkinje cell development [24]. In an autopsy study in early-onset SCA6, Purkinje cells were found to have reduced dendritic mass and spines, as well as reduced dendritic branching complexity [25,26]. Recent studies have shown that patients with *CACNA1A* mutations exhibit atrophy of the cerebellum during development, which is a recognizable neurodevelopmental disorder [27]. 

### 2.3. SCA14

Mutations of PKCγ cause SCA14 and in a mouse model of SCA14 expression of mutated PKCγ leads to dendritic abnormalities of Purkinje cells [28,29,30]. Increased PKCγ activity was identified as a negative regulator for Purkinje cell dendritic development [31]. Although many isoforms of PKC are expressed in the cerebellum, PKCγ is specifically and strongly expressed in Purkinje cells [32,33,34]. PKCγ expression is relatively low at birth and increases in the postnatal period [35,36]. PKCγ can be activated by binding of diacylglycerol (DAG) and Ca^2+^ [37]. In rat organotypic slice cultures, Purkinje cells were shown to grow increased dendritic trees with more ramified dendritic branches after treatment with a general PKC inhibitor, GF109203X [38]. Treatment of an inhibitor specific for conventional PKC isoforms, Gö6976 also promoted extensive branching of Purkinje cells [39]. In slice cultures from PKCγ deficient mice, Purkinje cells were shown to have expanded dendritic trees with an increased number of dendritic branching points compared to wildtype mice [39]. When Purkinje cells are treated with phorbol-12-myristate-13-acetate (PMA), an activator of PKC, a marked reduction of the dendritic trees was shown in either organotypic slice cultures or dissociated cerebellar cultures [28,30,38,39]. 

### 2.4. SCA15/16/29

Similar to SCA6 involving calcium channels, many genes of Group II SCAs caused by point mutations have been found to be involved in the regulation of the calcium equilibrium. SCA15 [40], also known as SCA16 [41], is caused by a heterozygous mutation in the *ITPR1* gene and by deletions involving the *ITPR1* gene. The *ITPR1* gene encodes inositol 1,4,5-trisphosphate (IP3) receptor type 1, mediating intracellular calcium release. The ITPR1 protein had a decreased expression in lymphocytes of the patient, suggesting a loss of function or a haploinsufficiency of ITPR1 protein [42,43]. SCA29 is also related to the *ITPR1* gene but has different single-nucleotide variants in *ITPR1*. The clinical features of SCA29 differ significantly from SCA15. SCA29 has non-progressive features and occurs congenitally, whereas SCA15 is a progressive cerebellar ataxia and occurs in adulthood [44,45]. The SCA29 missense mutations of ITPR1 protein are localized in the functional domain that refers to coupling or regulatory events and phosphorylation sites to influence the regulation of ITPR1 protein signaling [45]. Abnormal dendritic development of Purkinje cells is reported for cultured cerebellar cells from *ITPR1* knockout mice [46,47]. 

### 2.5. SCA41

The mutation Arg762His of TRPC3 protein has recently been identified to result in SCA41 [48,49]. To date, no SCA41 disease-associated mouse model has been reported, however, the *moonwalker* (Mwk) mouse mutant is caused by a different Trpc3 protein point mutation T635A and exhibits profound impairment of growth and differentiation of Purkinje cell dendrites [50]. The TRPC3 protein Arg762His mutation was shown to have a similar phenotype to the mouse Mwk *Trpc3* mutation in cellular experiments [48]. In addition, disrupted regulation of *TRPC3* has been reported in other SCAs. TRPC3 protein downregulation has been reported in Purkinje cells of a SCA1 mouse model before the onset of Purkinje cell degeneration [51]. Failure of TRPC3 protein phosphorylation by mutant PKCγ from SCA14 has been shown in cellular assays [52]. TRPC3 protein is mainly distributed in the cerebellar Purkinje cell layer during the stage of dendritic growth and functions in both dendritic growth and survival of Purkinje cells [50]. TRPC3 protein is highly expressed in the soma and dendrites of Purkinje cells during postnatal development of the cerebellum [50,53], and the high level of TRPC3 protein expression continues to the period of adulthood, suggesting that TRPC3 protein may also regulate the growth and refinement of dendritic trees of Purkinje cells in the cerebellar cortex during adulthood [50].

### 2.6. SCA44

SCA44 is caused by mutations of mGluR1 and missense mGluR1 mutants can result in increased receptor activity [54]. Activation of mGluR1 signaling has been reported in the *moonwalker* mouse model [50]. mGluR1, a subtype of the group I mGluRs, is most strongly expressed in Purkinje cells starting during the embryonic period [55,56,57,58]. Although no marked abnormality in cerebellar anatomy of mGluR1 deficient mice was found, the Purkinje cell dendritic arbors were found to be smaller and have a reduced complexity of Purkinje dendritic branches [59]. In dissociated cultures of rat cerebellar Purkinje cells and granule cells, pharmacological inhibition of mGluR1 by application of the subtype-selective antagonist of group I metabotropic glutamate receptors 7-(hydroxyimino)cyclopropa[b]chromen-1a-carboxylate ethyl ester (CPCCOEt) reduced the number of surviving Purkinje cells and the size of their dendritic arbors. These findings have been confirmed by rat *in vivo* experiments via local injections of LY367385, a highly selective and competitive mGluR1a receptor antagonist, mGluR1 antisense oligonucleotides, or systemic administration of CPCCOEt [60]. In contrast, in cerebellar organotypic slice cultures derived from P8 mouse pups and maintained for 12 days, pharmacological inhibition of mGluR1 by (RS)-alpha-methyl-4-carboxyphenylglycine (MCPG), a competitive metabotropic glutamate receptor antagonist, had an only minor negative effect on Purkinje cell dendritic morphology [61]. These studies indicate that mGluR1 signaling is essential for Purkinje cell growth and survival particularly at earlier developmental stages. At later developmental stages, mGluR1 signaling has an important regulatory function in the period of rapid dendritic expansion in cerebellar slice cultures. When mGluR1 signaling was induced by treatment with Dihydroxyphenylglycine (DHPG), a group I mGluR activator, a severe reduction of Purkinje cell dendritic growth was found [62].

### 2.7. SCA Pathogenesis Is Linked to Purkinje Cell Dendritic Development

The pathogenesis of SCA is still unknown and gain-of-function, dominant-negative-function and loss-of-function mechanisms have been proposed. However, none of them can adequately explain the pathology. For example, in SCA14, PKCγ knockout mice show relative normal cerebellar development and function making a simple loss-of-function explanation unlikely [63]. Indeed, inhibition of PKCγ activity by pharmacological inhibitors in cerebellar organotypic slice culture may even promote Purkinje cell dendritic development [39]. The toxic gain-of-function hypothesis has been proposed because aggregation of PKCγ was observed to exert a toxic effect on cells in some studies. For the PKCγ(H101Y) mutation, it has been reported that the PKCγ(H101Y) transgenic mouse exhibits an ataxic phenotype with altered morphology and loss of Purkinje cells. PKCγ(H101Y) protein leads to a decrease in the overall activity of the PKC enzyme [64]. In addition to a decrease of kinase activity of PKCγ mutations, several PKCγ mutants with increased kinase activity have also been found [52]. The PKC activator PMA does lead to a reduction in dendritic growth of Purkinje cells, suggesting that increased kinase activity may be an explanation for some of the cases. In the PKCγ(S361G) transgenic mouse model of SCA14, Purkinje cells exhibited severe morphological abnormalities that resembled the inhibition of dendritic growth induced by PMA treatment. These results support the concept that increased kinase activity of PKCγ is involved in the pathogenesis of SCA14 [28,30]. More recently, a PKCγ mutant has been reported to be caused by PKCγ(R76X), which produces a short peptide with a pseudosubstrate domain that served as a pan-PKC inhibitor. The PKCγ(R76X) peptide is thought to act in a dominant-negative manner by inhibiting PKC activity and causing the death of Purkinje cells [65]. A similar situation occurs in other types of SCAs, such as SCA44, and the physiological function of different mutants causing the disease protein may vary and have different effects on dendritic development of cerebellar Purkinje cell. 

Based on the above findings, in this manuscript we explore a possible link between molecules regulating Purkinje cell dendritic development and the pathogenesis of SCAs. This idea has been supported by some molecules, such as the RORα protein, which is not a causative gene of SCAs but plays an important role during the progression of disease. The expression of RORα protein is downregulated in Purkinje cells from the SCA1 mouse model and changes of Purkinje cell development mediated by the RORα protein determine disease severity of SCA1 [12,66]. These results suggest that mutations in or dysregulation of the molecules that play a role for Purkinje cell development may contribute to SCAs and cerebellar diseases.

## 3. Dysregulated Gene Expression in SCAs

The normal cellular pathway for growth, development and differentiation depends on accurate gene expression. Dysregulation of gene expression is an acknowledged characteristic of several SCAs and has been proposed to trigger the pathogenesis of SCAs [4,5]. Several mutant SCA causing proteins are recognized as key factors associated with the regulation of gene expression and contribute to gene regulation on the transcriptional level. In some representative types of SCAs caused by polyglutamines, such as SCA1 and SCA7, the causative proteins are ataxins, which interact with transcriptional regulators. Ataxin-1 can directly interact with Capicua, the mammalian homolog of *Drosophila* Capicua and modulate the repressor activity of Capicua in mammalian cells. However, in the Ataxin-1 with polyglutamine expansion, the binding to and the repressor activity of Capicua are changed. Capicua is a key transcriptional regulator and plays an important role in gene expression especially during developmental processes [6]. In SCA7, the Ataxin-7 protein plays a role for gene expression through its interaction with the co-activator SAGA complex, an extremely conserved complex involved in gene expression [7,8]. SAGA is influenced by the mutant Ataxin7 and clusters in a dysfunctional negatively charged SAGA complex, that is proposed to be involved in the pathogenesis of expanded polyglutamine diseases [9]. 

Some SCA causative genes are directly responsible for the regulation of gene expression. The TATA-Box binding protein (TBP) is a general transcription factor and mutants of TBP cause a CAG repeat expansion resulting in SCA17 [10]. TBP contains polyglutamine regions and these regions play a role in the ability of TBP to promote or inhibit transcription by interaction with regulators or by binding to different promoter areas. Mutations of TBP cause reduced affinity for binding to the relative region of DNA [11]. After completion of transcription as the first step of gene expression, protein synthesis in the next step involves essential enzymes called aminoacyl-tRNA synthetases. Importantly, mutation of genes encoding aminoacyl-tRNA synthetases can also cause cerebellar ataxia in a mouse model [67]. These studies suggest that mutated proteins of SCAs can affect gene expression directly or indirectly by changing the activity of signaling proteins resulting in a dysregulation of transcription. Many studies, therefore, have focused on the analysis of gene expression on the global transcriptional level by using animal or cellular models and aimed to identify genes with a strongly altered expression. These genes are expected to pinpoint the pathways associated with SCAs. 

## 4. Abnormalities of mGluR1 Signaling Associated with Pathogenesis of SCAs

Based on the global transcriptional data, several dysregulated molecules have been found from different mouse models with cerebellar ataxic phenotype, indicating potentially important pathways associated with pathogenesis of SCAs and cerebellar ataxia. For example, *staggerer* mice exhibit a characteristic severe cerebellar ataxia due to an underdeveloped cerebellar cortex and unaligned Purkinje cells [68]. Overlap based analysis of microarray data from the SCA1 mouse model [12] and *staggerer* mouse [13] was performed and mGluR1 was identified as a common molecule from overlapping the data of both mouse models. This molecule and associated signaling will provide a better understanding of the disease mechanisms of SCAs and cerebellar ataxia. 

A variety of different mouse models have been created referring to molecules of the mGluR1 signaling complex, including mGluR1, Gαq, PLC, PKCγ, ITPR1 and TRPC3 [43,69,70,71,72,73,74,75,76,77]. Some of these molecules are known to cause SCAs or other disorders relating to the cerebellum. For example, PKCγ mutants cause SCA14 [28,29,30] and PKCγ is downstream of mGluR1 signaling. SCA15 is caused by mutations of ITPR1 [40], another downstream molecule of mGluR1. In SCA1, mGluR1, ITPR1, PKCγ and Homer3 have been found to be downregulated on the transcriptional level [12,51,78] and for mGluR1 this reduction of expression has been confirmed on the protein level [79]. Disruption of mGluR1 has been reported in the mouse models of SCA3 [80] and SCA5 [18]. Mutations within *GRM1* coding for mGluR1 are relatively rare. Recently, SCA44 has been reported, with heterozygous dominant mutations in the *GRM1* gene showing typical phenotypes of SCA disease, but with different characteristics, possibly due to different functional changes in different mutants [54]. The function of an mGluR1 truncation mutation was tested by cellular experiments and this mutation resulted in a decreased receptor activity and decreased downstream target phosphorylation, suggesting that a loss of function of this mutation interferes with downstream signaling of mGluR1 [54]. These findings demonstrate an important role of mGluR1 signaling in Purkinje cells and show the relationship of altered mGluR1 signaling and SCA pathogenesis. 

Evidence for enhanced mGluR1 signaling is also present in different types of SCA. For example, elevated calcium is reported in Purkinje cells of the SCA2 mouse model caused by *ATXN2^Q58^* [81] and mutant ataxin2 can interact with IP3R [82]. Our previous study has reported increased IP3R1 expression (encoded by *ITPR1*, causative gene of SCA15 and SCA29) in an SCA14 mouse model [83]. The two other SCA44 causing mGluR1 missense mutations showed increased receptor activity compared to wild type mGluR1, suggesting that increased activity of mGluR1 leads to increased ligand sensitivity and ligand-independent activation, which is related to the fact that both missense mutations are closely located in the area of mGluR1 activation, resulting in excessive mGluR1 signaling by positive feedback with increased intracellular calcium levels [54]. The *moonwalker* (Mwk) mouse model with severe ataxia and abnormal Purkinje cell development, is caused by point mutations of *TRPC3* (the causative gene of SCA41). This mutant TRPC3 can activate the cation channel, downstream of mGluR1 signaling [50,84]. These studies suggest that an increased activity of the mGluR1 pathway might also be associated with pathogenesis in some types of SCAs. 

## 5. The Role of Recently Identified Dysregulated Molecules 

Researchers have used the strategy of overlapping microarray data from different types of SCAs. Recently, these mouse models have been used to identify some key molecules which could contribute to uncover potentially shared molecular mechanisms related to the pathogenesis of SCAs [14,85,86,87].

### 5.1. RGS8

RGS8 is dysregulated in SCA1, SCA2, SCA7 and SCA14, indicating a role in pathogenesis of SCAs [14,85,86,88]. RGS8 has been reported to be strongly expressed in rat cerebellar Purkinje cells and it appears to be enriched in brainstem and nucleus accumbens [89,90,91,92]. *RGS8* mRNA selectively interacts with ATXN2 and mutant ATXN2 reduced RGS8 expression in the SCA2 mouse model [86]. In a mouse model of SCA14 with increased PKCγ activity, RGS8 function has been studied in more detail. The increased RGS8 expression could partially counteract the negative effect of activated mGluR1 signaling during Purkinje cell development [85]. Since RGS8 is belonging to the R4 subfamily, its function is directly linked to Gq protein. RGS8 inhibits the M1 muscarinic acetylcholine receptor-Gq-mediated signaling in *Xenopus* oocytes [93] and has a strong inhibitory function for Gαq- and Gαi/o-dependent receptor activity [94]. However, RGS8 was also demonstrated to function via direct interaction with the relative receptor. RGS8 is able to interact with the third intracellular loop of melanin-concentrating hormone (MCH) receptor 1 (MCHR1) and inhibits the calcium mobilization induced by melanin-concentrating hormone [94]. Increased RGS8 expression through the inhibition of the MCHR1 signaling in the hippocampal CA1 region may be related to the antidepressant-like behavior of RGS8 transgenic mice [95]. Although the expression of RGS8 protein has been reported in the hippocampal CA1 region, RGS8 knockout mice have normal brain development and no major abnormalities in other organs [95,96]. RGS8 protein is also expressed in testis, but *RGS8* knockout mice are viable and fertile [96]. Electroconvulsive seizures in rats caused an increase in *RGS8* mRNA expression in the prefrontal cortex [97], suggesting a potential role for RGS8 in seizures. 

### 5.2. INPP5A

INPP5A protein is identified as a common molecule dysregulated in SCA1, SCA2, SCA7, SCA14 and SCA17 [14,85,86,87]. The molecule INPP5A is an enzyme of the inositol polyphosphate 5 phosphatase family. In cellular signaling, INPP5A functions as an enzyme that inactivates IP3 to terminate downstream signaling [98,99,100]. The absence of Inpp5a protein leads to progressive degeneration of Purkinje cells. In SCA17 knock-in mice, reduced Inpp5a expression was reported, which was associated with increased IP3 levels. Importantly, overexpression of *Inpp5a* gene causes a reduction of IP3 levels in the cerebellum and rescues the symptoms of Purkinje cell degeneration in SCA17 mice [87]. Similar to SCA2, overexpression of Inpp5a alleviates Purkinje cell degeneration in SCA2 mice [101]. 

### 5.3. STK17B

Down-regulated mRNA of *STK17B* is found in SCA1, SCA7 and SCA41 mouse models. In a recent study about *STK17B* gene function in Purkinje cells, STK17B signaling has been identified as a downstream effector of PKCγ. Reduction of STK17B protein is confirmed specifically in the Purkinje cells from SCA14 mouse models [14,102,103]. STK17B, also known as DAP kinase-related apoptotic kinase 2 (DRAK2), is located on chromosome 2 (2q32.3) and was first isolated from human placenta and liver cDNA libraries [104]. It belongs to the serine/threonine kinase family of the death associated proteins (DAP). *STK17B* gene is related to *STK17A* gene, also known as *DRAK1* gene, and both of them may constitute of a novel sub-family, which was originally thought to have the function of inducing apoptosis [104]. The STK17B protein structure includes an N-terminal autophosphorylation region and a C-terminal region with a nuclear localization signal. Another putative nuclear localization signal has been reported in the kinase domain [105]. STK17B has been relatively well studied in immunology as it is expressed in the immune system. The findings from the immune system may provide further insight for future studies on the function of STK17B in the brain. STK17B has been associated with calcium mobilization and homeostasis [105,106]. As the exact role of STK17B in brain is currently unclear, further studies would be helpful to better understand the function of STK17B in the nervous system in the future. STK17B protein has been reported to have a prominent expression in the brain, including the olfactory lobe, pituitary, superchiasmatic nuclei, ventricular zone and cerebellum [107]. Importantly, increased phosphorylation of STK17B protein causes negative effects on cerebellar Purkinje cell dendritic development. This negative effect can be partially rescued by a newly designed STK17B protein inhibitor Cpd16 [103]. These new findings give more insights for the treatment of SCAs. The new inhibitor could also be a potential drug for SCAs. 

**Figure 1:**
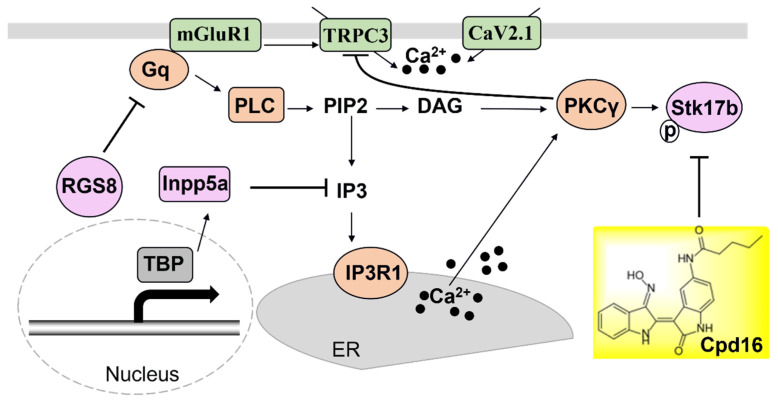
The recently reported molecules RGS8, Inpp5a and Stk17b are involved in the mGluR1-PKCγ signaling pathway in the cerebellum. A new drug, Cpd16 has been shown to work as inhibitor to Stk17b, which can regulate the downstream mGluR1 signaling pathway.

Taken together, these studies add new molecules associated with the alteration of a calcium channel and a compound to pharmaceutically manipulate mGluR1 signaling (Figure 1), which is thought to be an important factor in the pathology of SCAs. 

## 6. Shared mGluR1-PKCγ Signaling Pathway in SCAs

Since the different SCAs share the same cerebellar phenotypes, it is reasonable to assume that the underlying common pathogenic signaling could refer to the pathogenic features in the cerebellum, e.g., Purkinje cell degeneration. Through the review of recent studies of SCA mouse models, we summarize and find that mGluR1-PKCγ signaling is a common pathway that is dysregulated early in the onset of SCAs and is associated with Purkinje cell dendritic development. 

**Figure 2:**
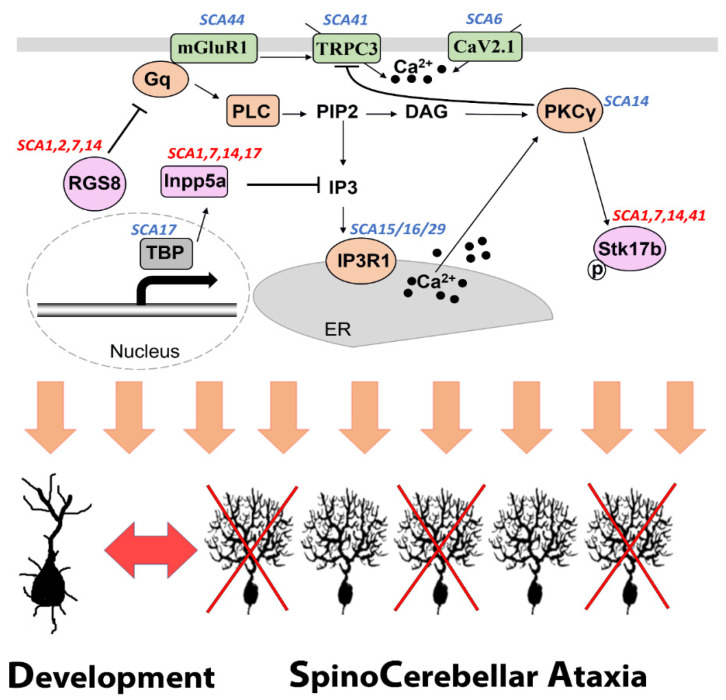
A shared mGluR1-PKCγ signaling pathway associated with SCA pathogenesis and Purkinje cell dendritic development. Several proteins which are components of the mGluR1-PKCγ signaling pathway are encoded by genes mutations of which are known to be causing SCA (labeled in blue). The common molecules RGS8, Inpp5a and Stk17b have been reported to be dysregulated in at least four different forms of SCA (labeled in red) and are directly involved in the mGluR1-PKCγ signaling pathway.

Dysregulated expression of common molecules is not only related to Purkinje cell dendritic development, but to the pathology of disease. This indicates similar signaling events which occur in the early stage of disease. For the group I type, e.g., SCA1, SCA2, most of their SCA genes are responsible for transcription [1]. A change of mGluR1-PKCγ signaling should be an indirect response during the early state of the disease. For the group II type, the SCA-genes are directly pointing to shared signaling or relevant downstream signaling, such as mGluR1, PKCγ, ITPR1, and TRPC3 protein [70,71,73,74,75,77]. Elucidating these shared pathways will help us to possibly modulate and monitor pathogenesis of different SCAs (Figure 2). 

Although the alteration of mGluR1-PKCγ signaling is thought to affect dendritic development of Purkinje cells and to occur much earlier than at disease onset, dendritic development of Purkinje cells may not be the direct cause of the adult ataxia phenotype. The cause of ataxia is thought to be related to changes in the firing pattern of mature Purkinje cells. For example, deletion of the SCA13-associated gene *KCNC3* affects the frequency of firing of Purkinje cells and increases the excitability of Purkinje cell dendrites [108]. Since mouse models of ataxia share the alteration of output signals in Purkinje cells, researchers have found that proper function of mGluR1 receptors and mGluR1 signaling are involved in the prevention of ataxia, which in turn suggests a link between the mGluR1 signaling pathway and ataxias. Further studies have shown that gene mutations related to mGluR1 or related signaling molecules cause the failure of climbing fiber maturation during the establishment of Purkinje cell innervation [109]. However, the suggested mGluR1 signaling pathway is certainly not able to explain the mechanism of all types of SCA. In a mouse model of SCA27, there was no significant change in the mGluR1 response, but the AMPA-mediated currents were impaired [110], suggesting that the mGluR1 pathway is not unique and that there are other potential signaling pathways that may cause SCAs. 

Recently, an adult-stage RNA profiling was presented in a study using the SCA2 mouse model, with the expectation of finding signaling pathways important for Purkinje cell degeneration. Inpp5a and RGS8 have also been identified as dysregulated molecules in this adult mouse study. In addition, there are also many molecules associated with calcium signaling, such as Camk2a and Camk4, which have been described as downstream candidate factors of mGluR1 signaling in synaptic function [111,112]. Although these molecules have not been identified in the developmental phase and few studies have reported functions of these molecules for dendritic development of Purkinje cells, it is worthwhile to investigate them to gain a deeper understanding of SCA pathogenesis. 

Serological testing using antibodies could help neurologists to diagnose cerebellar ataxias in clinical practice. Recent studies have shown that selective antibodies against molecules of the mGluR1 pathway are often present in patients with Autoimmune Cerebellar Ataxia. Importantly, many of these antigens are also associated with the pathogenesis of spinocerebellar ataxias [113,114,115]. This clinical evidence suggests that the mGluR1 signaling pathway may be a common pathophysiological mechanism not only in SCAs but also in other conditions with signs of cerebellar ataxia.

## 7. Conclusions and Outlook

The evidence of an association between Purkinje cell development and SCAs reveals an important role of molecules involved in Purkinje cell development and function and pathogenesis of SCAs. The molecules involved in mGluR1-PKCγ signaling are all strongly expressed in Purkinje cells [116,117]. Overlapping cerebellar pathogenic symptoms and abnormal Purkinje cell dendritic growth in SCAs accompany dysregulation of these common genes, suggesting the existence of shared cellular pathways linking multiple forms of SCAs [14,85]. In this review, we provide evidence that suggests that mGluR1-PKCγ signaling might be an important pathway shared in several different types of SCAs. In Table 1 we list those subtypes of SCA, for which abnormalities of signaling or changes of expression of important components of the mGluR1-PKCγ signaling pathway have been reported. Interestingly, this signaling pathway is associated with the recently identified common genes *STK17B*, *RGS8*, *INPP5A* [14,85,86,87,103]. Since by now there is no known efficient treatment strategy for SCAs, the design of new therapeutic strategies would be important which either suppress the progression of SCAs or at least alleviate the symptoms of the disease. STK17B is identified as a downstream mediator of mGluR1-PKCγ signaling. Cpd16, a new inhibitor STK17B, has been reported to partly rescue the phenotype of Purkinje cell abnormality [103,118]. It might be promising to study the utility of Cpd16 in SCA patients in the future. RGS8 is reported as a key molecule with dysregulated transcription in different SCA mouse models. RGS8 is also a kinase which locates upstream of mGluR1 signaling [85]. Strategies of designing drugs for RGS family members have been applied for cancer treatment [119,120]. Such drugs would also be promising candidates for targeting RGS8 in order to modulate mGluR1 signaling to improve the SCA associated disease symptoms in the future. 

In summary, despite a variety of different mouse models to investigate the pathology of SCAs, it is still unknown why different SCAs produce common deficits in the cerebellum. However, in many mouse models, abnormalities in genes involved in cerebellar developmental have been identified. In order to better understand this question, identifying potential dysregulated molecules to point out common signaling pathways was shown to be an efficient method. Future studies would need to combine diverse forms of SCAs using patient samples and mouse models in order to better define the common pathological mechanisms underlying diverse forms of SCAs. 

## Figures and Tables

**Table 1:** **The main SCA subtypes associated with mGluR1-PKCγ signaling**.

OMIM	Disease	Gene	Mutation	Protein	Inheritance	Clinical Phenotype	Model Used	References
164400	SCA1	*ATXN1*	CAG repeat expansion	Ataxin-1	Autosomal dominant	Ataxia, pyramidal signs, active reflexes, ophthalmoparesis	Mouse	[12,14,51,78,79]
183090	SCA2	*ATXN2*	CAG repeat expansion	Ataxin-2	Autosomal dominant	Ataxia, motor neuron involvement, hyporeflexia, dementia	Mouse	[81,82,86,101]
109150	SCA3	*ATXN3*	CAG repeat expansion	Ataxin-3	Autosomal dominant	Ataxia, motor neuron invovement, pyramidal features	Mouse	[80]
600224	SCA5	*SPTBN2*	Missense, deletion	β-III spectrin	Autosomal dominant	Cerebellar ataxia, facial myokymia, tremor	Mouse	[15,16,17,18]
183086	SCA6	*CACNA1A*	CAG repeat expansion	Caᵥ2.1	Autosomal dominant	Cerebellar ataxia, dysphagia, tremor, somatosensory deficit	Mouse	[19,20,21,22,23,24,25,26,27]
164500	SCA7	*ATXN7*	CAG repeat expansion	Ataxin-7	Autosomal dominant	Ataxia, retinal macular degeneration, dementia	Mouse	[7,8,14]
605361	SCA14	*PRKCG*	Missense, deletion	PKCγ	Autosomal dominant	Ataxia, tremor, dystonia, myoclonus	Mouse	[28,29,30]
606658	SCA15/16	*ITPR1*	Deletion	IP3R1	Autosomal dominant	Cerebellar ataxia, tremor, progressive	Mouse	[40,41,42,43]
607136	SCA17	*TBP*	CAG repeat expansion	TBP	Autosomal dominant	Ataxia, spasticity, dementia	Mouse	[10,11,87]
117360	SCA29	*ITPR1*	Missense	IP3R1	Autosomal dominant	Cerebellar ataxia, congenital non-progressive	Mouse	[45,46,47]
616410	SCA41	*TRPC3*	Missense	TRP-3	Autosomal dominant	Cerebellar ataxia	Mouse	[48,50]
617691	SCA44	*GRM1*	Missense, frameshift	mGluR1	Autosomal dominant	Ataxia, dysarthria, spasticity	Cell	[54]

Information was obtained from the references and Online Mendelian Inheritance of Men (OMIM) of SCAs.

## Data Availability

Not applicable.
